# Endovascular Embolization for Chronic Subdural Hematomas: A Literature Review of the Current Evidence

**DOI:** 10.7759/cureus.80898

**Published:** 2025-03-20

**Authors:** Andrés Sebastián Estrella López, Naomi Pauleth Espin Jiménez, Patricio Alejandro Montalvo Ramos, Gabriela Alejandra Castillo López

**Affiliations:** 1 Public Health, NeoScientia Consulting Group, Quito, ECU; 2 Rural Health, Ministerio de Salud Pública del Ecuador, Puyo, ECU; 3 Radiology, Medinuclear, Quito, ECU; 4 Outpatient Care, Integramed, Quito, ECU

**Keywords:** chronic subdural hematomas, current evidence, endovascular embolization, literature review, subdural hematomas

## Abstract

Chronic subdural hematoma (cSDH) primarily affects elderly patients along with those who have multiple health problems, while it develops when blood collects between the dura mater and the brain. The condition results in substantial disability while also causing surgical treatment failures in certain cases. The occurrence of cSDH becomes more frequent as people age beyond 65 years old, which establishes this condition as a major health issue. The standard surgical evacuation method for treating this condition shows limited success because patients experience frequent recurrences, which motivates researchers to study endovascular embolization as an alternative treatment option. The promising endovascular embolization technique uses middle meningeal artery (MMA) embolization to target the blood supply of the neo-membrane surrounding the hematoma, thus preventing rebleeding and reducing recurrence. The available data shows that embolization of the MMA leads to better recurrence prevention than conventional surgical procedures among patients who face restrictions for surgery because of multiple health issues. The review analyzes current research about MMA embolization safety together with its effectiveness and appropriate applications for cSDH treatment. The existing evidence about endovascular embolization as adjunctive therapy to surgical procedures such as burr hole drainage or craniotomy provides a significant impact over standalone treatment for complex and recurrent cSDH cases, reducing recurrence, rescue rates, and complications. However, endovascular embolization also shows better clinical outcomes in reducing these outcomes, but for less complex cSDH cases. There is unclear or inconclusive evidence for overall cSDH patients because current research lacks large multicenter randomized controlled studies together with stringent quality standards and contains various patient groups and minimal study durations. The procedure's effectiveness remains unclear because researchers have not determined the best patient selection criteria, the most suitable embolic agent, or the most economical approach.

## Introduction and background

Chronic subdural hematoma (cSDH) primarily affects elderly patients in their 70s and 80s, particularly those who have multiple health conditions, as blood accumulates between the dura and the brain. This accumulation leads to severe disability and often results in treatment failures. The pathophysiology of cSDH creates a neo-membrane that surrounds the hematoma, resulting in blood and cerebrospinal fluid accumulation. The symptom development occurs once compensatory mechanisms fail during this process [1]. The occurrence of cSDH rises significantly in people older than 65 years, with rates increasing from five per 100,000 in the general population to 58 per 100,000 in those aged 70 and above [2]. Research based on randomized trials demonstrates that cSDH leads to substantial mortality rates of 4% and morbidity rates of 11%. Medical health professionals recognize cSDH as a sentinel health event, as it leads to a reduction in substantial life expectancy compared to patients of similar age [3].

The surgical treatment of symptomatic cSDH through twist-drill or burr-hole craniotomy is considered both safe and effective and is a first-line treatment according to current literature. However, post-evacuation closed-system drainage has shown proven benefits [4]. The recurrence rate of cSDH remains a frequent concern at 11% based on evidence from observational studies. This recurrence leads to reduced functional outcomes and increased mortality risks, necessitating alternative treatment options [5]. The minimally invasive endovascular embolization technique through middle meningeal artery (MMA) embolization has established itself as a new treatment method to stop rebleeding and recurrence by blocking the blood supply to hematoma membranes [6].

Current clinical evidence about endovascular embolization treatment of cSDH focuses on both its effectiveness and safety, parameters, and patient choice criteria. Early research, together with later systematic reviews and meta-analyses, has shown that embolization is more effective at lowering recurrence rates than traditional surgery and maintains good safety results, particularly for patients who cannot undergo surgery [1,2,4]. However, the promising outcomes from these studies remain unclear, as varying study designs and patient groups fail to provide comprehensive answers about the optimal timing to perform embolization and its combination with other surgical treatments.

Purpose and scope

This review examines existing research to understand how endovascular embolization helps treat cSDH while indicating its advantages and weaknesses. It also proposes future recommendations for evidence-based practice. Understanding this emerging treatment approach will help medical professionals deliver better care to cSDH patients.

## Review

Pathophysiology

The primary characteristic of cSDH is blood accumulation between the dura mater and the arachnoid layer, which develops after minor head trauma. The condition affects elderly brain atrophy patients and those who take anticoagulant or antiplatelet medications. The initial bleeding from bridging veins causes acute bleeding, which starts the hematoma, but cSDH develops through multiple pathophysiological processes that create a neo-membrane and lead to vascular ingrowth and inflammation [7].

A traumatic event results in bridging vein rupture, which initiates the formation of cSDH within the subdural space. The blood leakage from this rupture results in the development of a hematoma. A foreign body response to the initial blood clot initiates an inflammatory cascade, which results in granulation tissue proliferation and the creation of a fibrous neo-membrane that surrounds the hematoma cavity. The neo-membrane develops from dural border cells, fibroblasts, and macrophages to produce angiogenesis in cSDH. The developing outer membrane becomes highly vascularized, but its immature blood vessels remain susceptible to rupture, which causes recurrent bleeding and enlarges the hematoma​ [8].

The recurrent nature of cSDH depends on the weak structure of newly formed vessels. Histopathological examinations reveal that newly formed vessels in the outer membrane of the hematoma demonstrate abnormal dilated structures with high permeability, which leads to continuous bleeding in the subdural space. The fragile new blood vessels in the hematoma area worsen the bleeding condition by causing repeated bleeding events that make cSDH treatment more challenging. The inflammatory conditions within the hematoma cavity activate pro-angiogenic factor release, which promotes new blood vessel formation and sustains bleeding and hematoma enlargement [9,10]. The pathophysiology can be assessed through visualization of hematoma as homogeneous hypodensity on CT scan.

The anatomical and physiological role of the MMA in cSDH

The MMA, a branch of the maxillary artery, plays a pivotal role in the development and recurrence of cSDH. The MMA provides blood supply to the dura mater while helping to develop the neo-membrane that surrounds the hematoma. The multiple branches of the MMA penetrate through the dura to supply blood to the outer membrane of the hematoma. The blood vessels inside the membrane serve as the main cause of persistent bleeding, which leads to cSDH recurrence [11].

Kim et al. (2024) also explained the neovascularization phenomenon in the outer membrane. The outer membrane of cSDH hematoma develops new blood vessels that stem from the MMA. The fragile nature of these vessels makes them susceptible to ruptures, which results in continuous bleeding inside the hematoma cavity. Angiographic studies demonstrate that MMA branches become dilated and create an abnormal vessel network, which sustains the hematoma in the outer membrane​. The MMA plays a vital role in the pathophysiology of cSDH recurrence because of its essential function in supplying blood to the outer membrane. The abnormal leaky vessels in the neo-membrane continue to bleed, which causes the hematoma to expand and results in repeated bleeding episodes [12].

Tudor et al. (2024) also show that MMA develops substantial vascular changes that support its role in cSDH pathogenesis. Selective angiography shows that the MMA develops pathological enlargement, which helps create abnormal vascular networks that penetrate the outer membrane of the hematoma​. The MMA demonstrates its central role in both the formation and recurrence of the hematoma by supplying most of the neo-vessels that develop in the outer membrane of recurrent cSDH patients [10].

Rationale

The MMA plays a vital role in cSDH pathogenesis, so its embolization has become an effective treatment choice for patients with recurrent or refractory cSDH. The main objective of MMA embolization treatment is to cut off blood flow to the neo-vessels in the outer membrane of the hematoma, which stops recurrent bleeding. MMA embolization blocks blood flow to the membrane through MMA branch occlusion, which prevents new vessel ruptures and allows the hematoma to absorb into the body [5,10,13].

A study demonstrates that MMA embolization proves effective for cSDH treatment, especially when treating patients with recurrent hematomas. Middle meningeal artery embolization procedures effectively decrease the likelihood of cSDH recurrence. The research by Ban et al. (2017) demonstrated that MMA embolization produced better results than standard surgical procedures because it led to a 1.4% recurrence rate compared to 27.5% for surgical patients​ [14]. The meta-analysis by Haldrup et al. (2020) systematically reviewed that recurrent cSDH patients who underwent MMA embolization developed only 2.4% of new events, but surgical treatments led to higher recurrence rates [5].

The primary indication for MMA embolization treatment is its ability to address the vascular abnormalities responsible for cSDH. The embolization procedure focuses on MMA branches that supply blood to neo-vessels because it addresses the primary cSDH recurrence factor by targeting vulnerable permeable vessels in the outer membrane. Research shows that the procedure successfully reduces hematoma size and controls further bleeding​ [5,10].

Technical aspects of endovascular embolization

Endovascular embolization of the MMA has become a new treatment for chronic subdural hematomas by presenting less invasive methods than traditional procedures, including burr hole drainage or craniotomy. This treatment blocks the blood supply to neo-vessels inside the hematoma membrane by focusing on the middle meningeal artery. Due to their weak structure, the blood vessels are easily ruptured, leading to further hematoma re-accumulation. The procedure blocks the arteries in the MMA, thereby blocking blood flow to new blood vessels, which subsequently ends bleeding and enables hematoma healing [15]. Medical practitioners have used multiple embolization methods to block MMA branches that feed the outer membrane of the hematoma. The embolic techniques utilize particle agents, liquid embolic agents, and coils, which provide distinguished mechanisms of action, precision levels, durability, and complexity of procedures [10, 16, 17].

Embolic Agents: Particle vs. Liquid

The MMA embolization procedures commonly employ two types of particle embolic agents, which include polyvinyl alcohol (PVA) and Embosphere (tris-acryl gelatin microspheres) [18]. The agents are engineered to block vessels and initiate an inflammatory response that results in vessel fibrosis. The PVA has been extensively used as a treatment for cSDH because of its established evidence. Surgeons or practitioners deliver these particles through a catheter to effectively block the larger branches of the MMA. The durability of embolization procedures using PVA particles can be reduced because vessel remodeling may lead to recanalization​ [19].

The advantages of Embosphere particles consist of their superior penetration capability within tiny vascular networks and their lower resistance to mechanical deformation when compared to PVA. The embolization properties of these embolic agents make them appropriate for distal embolization procedures. The procedure requires proper management of PVA and Embosphere particles to prevent catheter blockage, but both embolic agents can lead to catheter blockage if not properly managed [20].

Liquid Embolic Agent

The treatment of cSDH through MMA embolization now relies more frequently on liquid embolic agents, including Onyx, N-butyl cyanoacrylate (NBCA), and Squid. Liquid embolic agents provide better imaging visibility and longer-lasting vessel occlusion than particle embolics. Healthcare providers select Onyx among other co-polymeric formulations because its flexible properties create better delivery control and higher precision in embolization procedures. The main disadvantage of Onyx is its tendency to create imaging artifacts during extended injection periods, which makes it difficult to monitor embolization progress [21].

N-butyl cyanoacrylate functions as a cyanoacrylate-based liquid agent that turns into a solid form when it comes into contact with blood to create a permanent blockage. The medical community has achieved successful outcomes through its application in recurrent cSDH patients because it effectively decreases hematoma volume. The agent's tendency to stick to catheters becomes a major procedural challenge when multiple injections are needed because proper catheter management becomes essential​ [22].

Coil Embolization

The procedure of coil embolization serves as a treatment option for MMA embolization when used independently or in combination with particle or liquid agents. The procedure requires doctors to insert coils into the middle meningeal artery, which results in vessel blockage through mechanical means. The formation of a thrombus results in vessel fibrosis, causing long-term vessel blockage. Coil embolization serves as a treatment option for cSDH, but doctors use it primarily when complex vascular structures or dangerous ophthalmic artery anastomoses exist​ [10,23].

The use of coils offers protection against embolic materials migrating to non-target vessels because they prevent complications such as facial nerve damage and vision loss. The MMA branches require additional embolic agents when coil embolization fails to achieve complete vessel closure [10, 23].

Procedural Nuances

The success of MMA embolization for cSDH relies on selecting appropriate embolic agents, proper administration techniques, and complete knowledge of MMA anatomy. The literature identified various embolic agents summarized in Table 1, with their mechanism of action. Tudor et al. (2024) compared and analyzed the efficacy of each embolic agent, but the authors found evidence is limited due to small and low design evidence studies. Additionally, Tudor et al. (2024) also highlighted that registered trials with larger, more robust designs are currently undergoing, which may provide clear efficacy of each embolic agent for endovascular embolization [10]. Moreover, the microcatheter should be positioned at the main bifurcation point of the MMA, which gives rise to both frontal and parietal branches. The correct placement of the microcatheter at the main bifurcation of the MMA serves two essential purposes: it enables complete vessel blockage and avoids accidental embolization of unintended vessels. [10].

**Table 1:** A summary of embolic agents

Embolic agent class	Embolic agent	Mechanism of action
Particle agent [10]	Polyvinyl alcohol (PVA)	Adhered into the wall of the vessel with subsequent activation of coagulation cascade and vessel necrosis
	Embosphere(tris-acylgelatin)	Similar inflammatory reaction mechanism to PVA particles
	Gelatin sponge	Hemostatic embolic agent; provides a physical matrix for clot and granulation tissue formation
Liquid agent [10]	Onyx (ethylene vinyl alcohol) copolymer dissolved in dimethyl sulfoxide)	External-to-internal precipitation solidifies upon contact with the blood, creating a spongy, coherent embolus
	N-butyl-2-cyanoacrylate (NBCA)	Polymerization
	Lipidiol	Deposition
	Squid (ethylene vinyl alcohol copolymer and dimethyl sulfoxide)	External to internal precipitation
	Precipitating hydrophobic injectable liquid (PHIL)	External to internal precipitation
Coils [10]	Coil-only embolization	Stasis-induced thrombus formation and organization, myofibroblast invasion, and collagen deposition
	Coil and particle /liquid embolization	Coil-induced thrombus formation and extracellular matrix deposition generally follow the initial particle/liquid embolization-associated mechanism

The main difficulty during MMA embolization procedures arises from non-target embolic events that occur when using liquid embolic agents, which tend to flow into unintended vessels. The use of microcatheter wedging techniques and the dual-embolization method, combining spherical and liquid agents, reduces the possibility of non-target embolization [10, 13].

The surgeon must evaluate the MMA's branching pattern variations with great care. Patients with anatomical anomalies, such as ophthalmic or petrosal artery branches, risk complications when these vessels are embolized by mistake [13]. The treatment requires individualized strategies that combine coil embolization with other embolic agents when necessary​. Therefore, middle MMA stands as a promising therapeutic option for chronic subdural hematoma patients because it provides minimally invasive care instead of surgical interventions. Multiple factors, including patient vascular structure, hematoma dimensions, and comorbid medical conditions, determine which embolic agent will be selected between particles, liquids, and coils. Surgeons tend to choose liquid embolic agents, including Onyx and NBCA, because of their excellent embolic control and durable effects, but particle agents, including PVA and Embosphere, maintain significance because of their proven safety and effectiveness records [10]. Additional high-quality randomized controlled trials (RCTs) should be conducted to improve embolic agents and procedural techniques for cSDH treatment through MMA intervention according to the literature.

Clinical outcomes and safety

Middle meningeal artery embolization serves as a successful non-invasive medical procedure to treat cSDH. A systematic analysis, together with a meta-review of 20 studies following 1,416 patients, demonstrated the results of middle meningeal artery embolization and traditional cSDH treatment procedures. The collected data show that MMA embolization offers multiple advantages, such as lower recurrence rates, reduced surgical interventions, and fewer treatment-related complications, thus becoming an attractive alternative to already established treatments ​[24].

Efficacy of MMA Embolization

The recurrence rate for patients who received MMA embolization reached 4.8% (95% confidence interval (CI) 3.2% to 6.5%), while the conventional management group experienced a recurrence rate of 21.5% (95% CI 0.6% to 42.4%). The recurrence rate data matches previous research, which shows MMA embolization works to stop hematoma reaccumulation, especially in patients with recurrent bleeding. The odds ratio (OR) for recurrence with MMA embolization reached 0.15 (95% CI 0.03 to 0.75), which showed a statistically significant decrease compared to conventional management (p=0.02) ​[24].

The embolization group required surgical intervention less frequently than the conventional management group, as the embolization cohort experienced a surgical rescue rate of 4.4% (95% CI 2.8% to 5.9%) while the conventional management cohort experienced a rate of 16.4% (95% CI 5.9% to 27.0%). The surgical rescue rate with MMA embolization showed an OR of 0.21 (95% CI 0.07 to 0.58), which demonstrated a substantial decrease in subsequent surgical procedures (p=0.003)​ ​[24].

Safety and Complications

The pooled incidence of complications during hospital stays for patients who underwent MMA embolization reached 1.7% (95% CI 0.8% to 2.6%), while the conventional management group experienced 4.9% (95% CI 2.8% to 7.1%) complications. The analysis shows that MMA embolization leads to fewer complications, yet the difference between groups was not statistically significant (OR=0.78 (95% CI 0.34 to 1.76), p=0.55). Previous research supports these findings by showing MMA embolization produces minimal complications that are lower than those observed in traditional surgery ​[24]. The pooled statistics at a 95% CI are mentioned in Table 2.

**Table 2:** Pooled statistics illustrating clinical efficacy and outcome comparison of endovascular embolization with conventional treatment based on current literature evidence at a 95% confidence interval (CI).

Parameters	Endovascular embolization	Conventional/Surgical treatment
Recurrence rate [24]	4.8% (95% CI 3.2% to 6.5%)	21.5% (95% CI 0.6% to 42.4%)
Rescue rate [24]	4.4% (95% CI 2.8% to 5.9%)	16.4% (95% CI 5.9% to 27.0%)
Complication [24]	1.7% (95% CI 0.8% to 2.6%)	4.9% (95% CI 2.8% to 7.1%)

Comparative effectiveness as standalone versus adjunct therapy

Evidence indicates that middle meningeal artery embolization stands as a promising treatment method for cSDH, working alone or alongside surgery, mainly for patients with recurrent or treatment-resistant cases. Optimal patient care depends on understanding the distinct results that arise from either using MMA embolization independently or in combination with the surgical evacuation of cSDH.

Outcomes of MMA Embolization as Standalone Therapy

Recurrence rates: The use of stand-alone MMA embolization demonstrates its ability to decrease the risk of cSDH recurrence in affected patients. Ban et al. (2017) showed that patients receiving MMA embolization treatment alone achieved a 2.2% recurrence rate for symptomatic cases but the conventional treatment group experienced a recurrence rate of 27.5% [14]. Middle meningeal artery embolization proves effective because it stops recurrent bleeding from fragile neo-vessels that form inside the hematoma membrane. Medical microspheres demonstrate a 4.8% recurrence rate for treating recurrent cSDH patients based on Haldrup et al.'s (2020) meta-analysis [5].

Rescue rates: Patients undergoing embolization therapy needed surgical intervention much less often than those handled through standard treatment approaches. The study by Ban et al. (2017) showed that embolization treatment led to a rescue rate of 1.4% in cSDH patients but standard treatment resulted in 18.8%. The effectiveness of MMA embolization as a treatment for recurrent cSDH provides extended solutions to patients who suffer from this condition [14].

Safety and complications: The standalone MMA embolization procedure shows positive safety results in its operational characteristics. Ban et al. (2017) reported that embolization patients remained complication-free but the conventional treatment group developed complications in 4.3% of cases [14]. The patients treated with MMA embolization according to Kim et al. (2017) showed no major complications during their procedure. The data indicates that MMA embolization serves as a safe surgical intervention for cSDH patients because it produces minimal complications during and after the procedure [12]. The clinical outcomes of endovascular embolization as standalone therapy statistics are compared in Table 3.

**Table 3:** Endovascular embolization as a stand-alone therapy

Parameters	Endovascular embolization	Conventional/Surgical treatment
Recurrence rate [14]	2.2%	27.5%
Rescue rate [14]	1.4%	18.8%
Complication [14]	0% (no complication reported)	4.3%

Outcomes of MMA Embolization Combined with Surgical Evacuation

Recurrence rate: The treatment of large symptomatic hematomas requires a combined approach of MMA embolization with surgical evacuation through burr hole drainage or craniotomy. Scientific evidence shows that the combined procedure leads to substantial reductions in patients experiencing new bleeds. The combination of MMA embolization treatment with burr hole drainage prevented 91% (9% recurrence rate) of patients from needing surgical intervention for recurrence based on Kim et al. (2017) [12]. Standard surgical procedures benefit from the additional preventive effect when combined with MMA embolization treatment.

Rescue rates: Surgeons combine embolization with surgical procedures to considerably lower the need for future surgical interventions. Mino et al. (2010) studied patients undergoing combined MMA embolization treatment with burr hole drainage or craniotomy therapy, which decreased their recurrence rate to 3.8% as compared to the 33.3% recurrence rate for the surgical group only. The research shows that MMA embolization serves as an extra preventive method to stop blood from reaccumulating [25].

Complications: When MMA embolization techniques are combined with surgical procedures, they produce complication rates that match those observed in standard surgical procedures. The complication rates from surgical procedures combined with embolization matched traditional surgical complication rates, according to Kim et al. (2017) [12]. According to Haldrup et al. (2020), their study demonstrated that combined embolization surgery generated similar complication rates to standard surgical procedures. Middle meningeal artery embolization together with surgical intervention provides a secure treatment method for complex cSDH by eliminating the introduction of new procedural hazards [5]. The detailed comparative endovascular embolization as adjunctive therapy showed better comparative clinical outcomes for cSDH patients presenting with complex presentation (Table 4).

**Table 4:** Endovascular embolization as adjunct with surgical treatment

Parameters	Endovascular embolization with surgery	Surgical treatment alone
Recurrence rate [12]	9% (91% prevention)	33.3%
Rescue rate [25]	3.8%	33.3%
Complication [5]	Similar to the standard surgical complication rate	Standard surgical complication rate

The treatment of cSDH requires standalone MMA embolization or surgical evacuation with MMA embolization, but the selection depends on patient characteristics and hematoma complexity. Middle meningeal artery embolization functions as an important preventive method against bleeding recurrence in basic situations, but the combined approach delivers optimal benefits for treating resistant or recurrent cases. Therefore, factors related to patient characteristics (age, comorbidities), medication used (antiplatelets), and hematoma complexity (size, location, and unilateral or bilateral presentation hematomas) of cSDH must be considered while making decisions regarding treatment.

Age and comorbidities: Chronic SDH patients' risk factors are hypertension, diabetes, stroke, and liver dysfunction, which increase the incidence of cSDH. Patients who use anticoagulant or antiplatelet medications face increased risks of both recurrence and chronic subdural hematoma development. Research demonstrates that MMA embolization provides exceptional benefits to patients undergoing advanced medical procedures. Mino et al. (2010) established that MMA embolization produced beneficial clinical results for elderly patients with multiple health conditions, preventing them from undergoing invasive surgical procedures [25]. Middle meningeal artery embolization provides an effective reduction in recurrence rates for patients who need to avoid general anesthesia because of existing medical conditions.

Coagulopathies and antiplatelet use: Patients who have coagulation disorders or take anticoagulant medications experience higher chances of cSDH recurrence because the delicate new blood vessels in the outer membrane of cSDH can easily break. MMA embolization serves as a minimally invasive therapeutic approach for these patients because it reduces bleeding hazards without requiring major surgical interventions. The clinical outcomes from MMA embolization proved superior for patients on anticoagulants or antiplatelet medications because it decreased recurrence rates more effectively than surgical treatment methods, according to Kim et al. (2017) ​[12].

Size and location of hematoma: The therapy of surgery combined with MMA embolization creates the most effective treatment for large cSDH, which produces significant mass effects and is located in areas that surgery struggles to reach. Burr hole drainage or craniotomy must be used together for symptomatic large hematomas because individual procedures alone fail to achieve sufficient results and increase the risk of recurrence. The combination of MMA embolization with surgical evacuation produces significant recurrence prevention because embolization stabilizes the outer membrane's vascular fragility and surgical evacuation removes the hematoma's mass effect. Harldrup et al. (2020) also found that patients diagnosed with large-sized cSDH showed better recovery rates when treated with endovascular embolization, with controlled bleeding during the procedure [5].

Bilateral or multilocular hematomas: The treatment of bilateral or multilocular hematomas achieves its best results through the combination of MMA embolization with surgical evacuation because these complex vascular networks tend to recur often. Medical research indicates that multiple vascularized layers in these hematomas lead to higher recurrence rates when standard surgical drainage procedures are performed. The embolization procedure interrupts blood flow to all neo-vessels to reduce the risk of hematoma re-accumulation in these patients [26].

Organized cSDH: The development of fibrous membranes in organized cSDH makes MMA embolization ineffective because it increases the risk of bleeding returning. The medical treatment of organized hematomas demands craniotomy surgery to remove the outer membrane because extensive medical procedures become necessary. Middle meningeal artery embolization serves as a supplementary treatment method that medical professionals use to stop recurrence after operating on organized hematomas. Postoperative MMA embolization therapy in combination with craniotomy procedures demonstrated enhanced treatment outcomes for organized cSDH patients based on Matsumoto et al. (2017) [27].

Middle meningeal artery embolization functions as a beneficial medical procedure for treating patients with smaller unilateral non-organized hematomas and patients who face high surgical risks. Middle meningeal artery embolization with surgical evacuation provides the best possible outcomes for treating large, bilateral, multilocular, or organized hematomas. A medical background of the patient, age, and hematoma complexity should guide healthcare providers in selecting proper treatment methods. Medical embolization stands as an effective therapeutic intervention for treating both standard cases of cSDH and complex recurrent cSDH lesions.

Inconsistencies in study designs and findings

The existing research on MMA embolization for cSDH contains conflicting information because various studies employ distinct patient groups and research methodologies. The positive results and low recurrence data demonstrated by studies exist within retrospective and limited sample size frameworks ​[1,5,11,13,15]. The use of different embolic agents such as PVA particles, NBCA, and coils in various studies introduces treatment outcome variability. The diverse embolic agents used in treatment make it difficult to establish which agent provides superior results and suggest that the choice of embolic material affects treatment outcomes. Tudor et al. (2024) also highlighted and identified prospective registered trials, which may address and ensure the validity of findings through their clinical trials related to each agent used in MMA embolization [10].

Studies that combine MMA embolization with surgical evacuation show different recurrence rates in their results. The combination of MMA embolization with surgery achieved a 91% success rate in preventing reoperation for recurrent cSDH according to Kim et al. (2017)​ [12]. Matsumoto et al. (2017) discovered that MMA embolization with surgical intervention showed no better cure rates than burr-hole irrigation alone for refractory cSDH​ [27]. The variable treatment results from MMA embolization indicate that its effectiveness depends on multiple factors, including the complexity of the hematoma, patient health conditions, and the specific procedure performed.

Controversies in the efficacy and indications of MMA embolization

The main point of disagreement about MMA embolization for cSDH centers on its appropriate use between initial and repeated cases. According to Haldrup et al. (2020) and Mino et al. (2010), MMA embolization shows high efficiency in preventing recurrent cSDH, but experts debate using MMA embolization as a first-choice treatment for cSDH [5,25]. The research by Ban et al. (2017) shows MMA embolization works to prevent recurrence in primary cSDH patients, but some experts advocate for burr hole drainage as the primary treatment option [14].

Nearly equal amounts of medical controversy exist about employing MMA embolization independently of surgery and integrating its usage with surgical interventions. The combination of embolization with surgical drainage for recurrent or complicated hematomas shows beneficial results according to Kim et al. (2017) and Mino et al. (2010) [14,25] but standalone embolization produces similar outcomes in simpler cases. Medical management experts disagree about whether the embolization of MMA should stand by itself or require surgical assistance to properly control cSDH pathophysiological mechanisms [5].

Limitations

Multiple design weaknesses in MMA embolization studies for cSDH negatively impact the quality of available evidence. The main drawback stems from the absence of RCTs in the research. Studies on MMA embolization usually depend on retrospective data or involve small numbers of patients, which creates doubts about researcher selection preferences and omitted variables.

The patient populations across different studies exhibit diverse characteristics, which creates a limitation for the research. The research design includes elderly patients with coagulopathies in some studies, but other studies examine younger patients with less severe cSDH cases. The inconsistent patient characteristics across studies create challenges for determining how well the research findings can be applied to different groups of patients.

The research studies present inconsistent time periods for patient follow-up. The studies by Haldrup et al. (2020) maintain short follow-up durations between six weeks and several months, thus preventing an assessment of extended outcomes or recurrent patterns after treatment completion [5]. The long-term effectiveness of MMA embolization depends on obtaining extensive data to determine the persistence of its benefits when re-bleeding may happen months after the initial procedure.

Unresolved questions

Multiple existing questions persist regarding how MMA embolization should ideally be utilized for the treatment of cSDH. Scientists need to determine how durable MMA embolization treatments will be for patients experiencing cSDH recurrence. The preliminary results show good success with low recurrence rates plus small numbers of complications during early follow-up periods, but data about the technique's extended-term effectiveness remain limited. The effectiveness of MMA embolization for preventing recurrence is uncertain; will it maintain its benefits throughout multiple years after the treatment procedure? Further research must track patients through multiple years to resolve this question.

The choice of embolic agent remains an unresolved issue when performing MMA embolization procedures. Research using PVA particles and NBCA and coils as embolic agents has not established which material leads to superior clinical results. The selection of embolic materials affects both the length of treatment time and procedural risks and treatment effectiveness. A direct comparison between different embolic agents in large populations would result in more reliable recommendations.

Surrounding cost-effectiveness analysis for MMA embolization lacks proper examination in existing medical literature. Healthcare expenses likely increase when patients undergo this minimally invasive procedure because it demands specialized equipment along with expert personnel. The cost-effectiveness analysis of MMA embolization against traditional treatments, including burr hole drainage and craniotomy, must be established to determine its practical use in medical settings.

Gaps in the literature and future recommendations

The current literature lacks essential information about MMA embolization treatment for cSDH. The available RCT data for MMA embolization are insufficient because there are few large-scale multi-center trials conducted with proper experimental methods. Complete evaluation of MMA embolization requires large RCTs with proper design to compare its long-term results with traditional subdural hematoma (SDH) treatment methods.

The research lacks sufficient studies investigating MMA embolization treatment for primary cSDH cases, despite existing studies focusing on recurrent and refractory cSDH cases. The clinical applications of MMA embolization as an alternative to burr hole craniotomy for primary cSDH management would become clearer through understanding its effectiveness in this context.

The scientific basis for how MMA embolization stops recurrence remains unclear to medical experts. The proposed mechanism of embolization involves interrupting blood flow to neo-vessels inside the hematoma membrane, but additional evidence-based research is required to understand this process and determine predictive factors for procedure success or failure.

## Conclusions

The existing evidence about endovascular embolization as adjunctive therapy to surgical procedures such as burr hole drainage or craniotomy has a significant impact on standalone treatment for complex and recurrent cSDH cases, reducing recurrence, rescue rates, and complications. However, endovascular embolization also shows better clinical outcomes in reducing these outcomes for less complex cSDH cases. There is unclear or inconclusive evidence for overall cSDH patients because current research lacks large multicenter randomized controlled studies, together with stringent quality standards, and contains various patient groups and minimal study durations. The procedure's effectiveness remains unclear because researchers have not determined the best patient selection criteria, the most suitable embolic agent, or the most economical approach. Long-term prospective investigations with appropriate designs will help address knowledge gaps regarding MMA embolization usage and define appropriate clinical cases for cSDH treatment.
